# Chemotherapy can induce weight normalization of morbidly obese mice despite undiminished ingestion of high fat diet

**DOI:** 10.18632/oncotarget.14576

**Published:** 2017-01-10

**Authors:** Cheryl E. Myers, Dominique B. Hoelzinger, Tiffany N. Truong, Lindsey A. Chew, Arpita Myles, Leena Chaudhuri, Jan B. Egan, Jun Liu, Sandra J. Gendler, Peter A. Cohen

**Affiliations:** ^1^ Department of Biochemistry and Molecular Biology, Mayo Clinic, Scottsdale, AZ, USA; ^2^ Department of Immunology, Mayo Clinic, Scottsdale, AZ, USA; ^3^ Center for Individualized Medicine, Mayo Clinic, Scottsdale, AZ, USA; ^4^ Department of Hematology and Oncology, Mayo Clinic, Scottsdale, AZ, USA

**Keywords:** obesity, chemotherapy, proton leak, preadipocyte, lipogenesis

## Abstract

Morbidly obese patients who accomplish substantial weight loss often display a long-term decline in their resting metabolism, causing even relatively restrained caloric intake to trigger a relapse to the obese state. Paradoxically, we observed that morbidly obese mice receiving chemotherapy for cancer experienced spontaneous weight reduction despite unabated ingestion of their high fat diet (HFD). This response to chemotherapy could also be achieved in morbidly obese mice without cancer. Optimally dosed methotrexate (MTX) or cyclophosphamide (CY) enabled the mice to completely and safely normalize their body weight despite continued consumption of obesogenic quantities of HFD. Weight reduction was not attributable to decreased HFD intake, enhanced energy expenditure or malabsorption. MTX or CY dosing significantly depleted both adipose tissue and preadipocyte progenitors. Remarkably, however, despite continued high fat feeding, a compensatory increase in hepatocyte lipid storage was not observed, but rather the opposite. Gene microarray liver analyses demonstrated that HFD mice receiving MTX or CY experienced significantly inhibited lipogenesis and lipid storage, whereas *Enho* (energy homeostasis) gene expression was significantly upregulated. Further metabolic studies employing a human hepatocellular line revealed that MTX treatment preserved robust oxidative phosphorylation, but also promoted mitochondrial uncoupling with a surge in proton leak. This is the first report that certain optimally dosed chemotherapeutic agents can induce weight loss in morbidly obese mice without reduced dietary intake, apparently by depleting stores of adipocytes and their progenitors, curtailment of lipogenesis, and inconspicuous disposal of incoming dietary lipid via a steady state partial uncoupling of mitochondrial oxidative phosphorylation.

## INTRODUCTION

Obesity affects more than one-third of the adult population in the United States, and is identified as a major global health and economic concern by the World Health Organization [1, 2]. Excessive body fat is considered a negative independent risk factor for a number of life-altering diseases including metabolic syndrome, cardiovascular disease, type-2 diabetes, nonalcoholic fatty liver disease and cancer [3, 4]. An additional dilemma is that when morbidly obese patients achieve impressive weight loss through dietary restraint and intensive exercise, they often display a poorly understood long-term reduction in their resting metabolic rate which causes even relatively moderate caloric intake to provoke recurrent obesity [5].

Apart from these concerns, there is a tendency for obese patients with malignancies to be undertreated with chemotherapy, due to apprehension about their ability to tolerate its comorbidities [6–9]. Recent American Society of Clinical Oncology (ASCO) guidelines suggest that chemotherapy dosing for obese patients should be calculated using actual body weight rather than ideal weight or capped dosing, unless there are other complications [6]. These recommendations are based on meta-analyses of obese cancer patients, identifying studies published in English between 1996 and 2010. Most patients had breast, ovarian, colon or lung cancers treated with standard-of-care chemotherapy. Such analysis revealed no increased chemotherapy-related toxicities compared to non obese patients when dosing was based on actual body weight, and therapeutic outcomes to chemotherapy were comparable in the two cohorts. It was acknowledged, however, that such findings might not extrapolate to clinical trials including novel therapeutics [6]. Additional studies in mice suggest that persistent high fat diet (HFD) and untreated insulin resistance can modestly accelerate tumor progression [10–12], but these studies were performed in the absence of chemotherapy.

Our own effort to observe whether chemotherapy's anti-tumor efficacy varied for normal versus obese mice evidenced no significant differences, consistent with the ASCO analysis. Surprisingly, however, the weekly weighing of mice to adjust their chemotherapy doses revealed that chemotherapy-treated obese mice had an unsuspected and remarkable tendency for weight reduction despite their continued avid ingestion of HFD. This paraphenomenon was also observed in non-tumor-bearing obese mice, with two chemotherapeutic agents, cyclophosphamide and methotrexate, proving to be particularly well tolerated during weeks of treatment, facilitating complete weight normalization despite unabated HFD intake. Explanations such as malabsorption, decreased caloric intake, increased energy expenditure, or ketotic excretion of calories were effectively ruled out as explanations for weight normalization. Instead, a pronounced loss of lipid stores associated with a log-fold cytoreduction of adipocyte progenitors was evident. Remarkably, despite continued high fat feeding, a compensatory increase in hepatocyte lipid storage was not observed during chemotherapy, but rather a stable background increase in uncoupled oxidative phosphorylation which could inconspicuously dissipate calories through a steady-state proton leak.

## RESULTS

### Whereas chemotherapy's antitumor impact is unaffected by diet-induced obesity (DIO), chemotherapy itself can induce a trend towards weight normalization in obese mice

**Figure 1:**
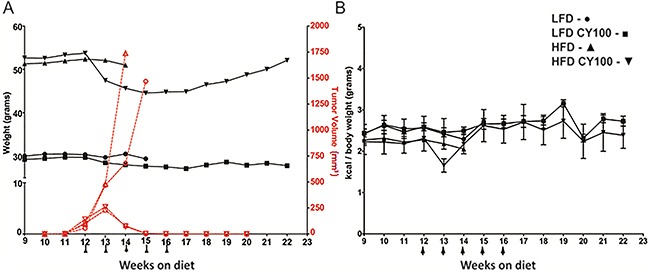
MC38 tumor growth and chemotherapy response in HFD mice vs LFD mice **A**. Growth rates of MC38 colorectal tumors implanted in syngeneic B6 mice after 10 weeks on a continuing LFD or HFD diet were measured weekly, with or without cyclophosphamide 100 mg/kg (CY100) beginning at week 12 (LFD - ○, LFD CY100 - □, HFD - D, HFD CY100 - — tumor volumes reflected on right red axis). Weekly body weights (LFD - ·, LFD CY100 -■, HFD - ▲, HFD CY100 - ▼ reflected on black left axis) were used to adjust CY100 dosing to current actual body weight, with CY100 given i.p. weekly for 5 cycles (each CY dose depicted as an arrow “↑”). Each treatment condition consisted of 3 to 5 mice with 2 biological replicates. For HFD vs LFD mice not receiving CY there were no significant differences in tumor progression requiring euthanasia (median day of euthanasia, HFD day 30.3 ±4.0 vs LFD day 31.4 ± 6.9, *p*=0.3 by *t*-test). Cure rates for LFD vs HFD mice receiving chemotherapy were identical (both 100% cured, Fisher's exac*t*-test *p*=1.0), with superimposable rejection kinetics. However, HFD mice receiving CY displayed progressive loss of weight at weeks 13 through 17, which reversed when CY was discontinued. Error bars are omitted in (A) to make the individual treatment events easier to discern. **B**. Kilocalories consumed per body weight. No significant differences were seen among groups except for HFD vs HFD+CY after the first week of CY (week 13, HFD 2.2 ± 0.01 kcal/gm vs HFD+CY 1.7 ± 0.2 kcal/gm, *p*<0.02 by *t*-test). After 16 weeks only the groups receiving CY survived and continued to be monitored. As in **(A)** each treatment condition consisted of 3 to 5 mice, with 2 biological replicates.

Six-week old C57BL/6 male mice were randomly assigned to either a 60% lard HFD or a 10% low fat diet (LFD), remaining on the same diet at all stages of treatment. Eight weeks later, mice were individually housed to precisely monitor food intake. At 10 weeks on diet, 5×10^5^ MC38 colorectal tumor cells were injected subcutaneously. Two weeks after implantation, MC38 tumors averaged 6mm in diameter, at which time cyclophosphamide 100 mg/kg (CY100) was given to the chemotherapy cohorts for 5 weekly treatments (Figure 1A, arrows). Dosing was recalculated weekly and was based on the concurrent actual body weight.

HFD-fed mice were morbidly obese and weighed significantly more than LFD-fed mice prior to chemotherapy (average 52±5.8g, 30±2.1g respectively) (Figure 1A, black lines). Tumor-challenged obese HFD mice not receiving chemotherapy averaged a slightly faster tumor progression than LFD mice but this was not significantly different (Figure 1A). In contrast, CY100 treatment of mice, whether on HFD or LFD, resulted in kinetically indistinguishable complete and durable tumor regressions by the end of three weekly chemotherapy cycles (Figure 1A). Therefore, obesity did not detectably modulate the anti-tumor impacts of CY100.

Nonetheless, during chemotherapy, we observed strikingly different weight trends in the two dietary cohorts. Prior to chemotherapy, HFD-fed obese mice consumed approximately 10% fewer kcal than non-obese LFD-fed mice (normalized to body weight, Figure 1B). HFD-fed obese mice treated with CY100 also displayed a transient drop in food consumption during the first cycle of chemotherapy, but their HFD intake quickly rebounded during continued cycles of CY100 (Figure 1B). Notwithstanding their robust ingestion of the HFD, obese mice receiving CY100 showed a significant loss of total body weight during the remainder of the treatment period (weeks 13-16). Interestingly, after chemotherapy ceased, HFD mice continued to display the same caloric intake per body weight but began gradually to regain body weight, once again trending towards morbid obesity (Figure 1A).

### Weight normalization observed in non-tumor bearing HFD-fed mice treated with chemotherapy does not reflect decreased caloric intake, increased energy expenditure, or malabsorption

**Figure 2:**
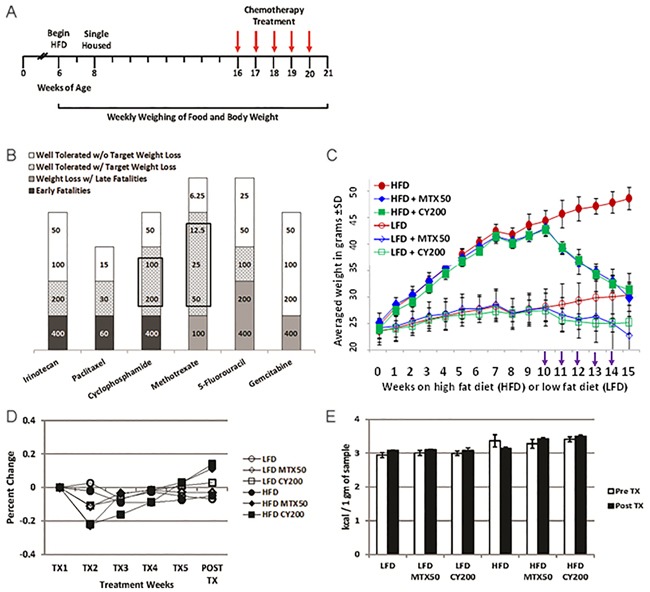
Intermediate chemotherapy dosing fully normalizes body weight of non tumor-bearing obese mice **A**. Schematic for non tumor-challenged LFD and HFD mice treated with chemotherapy in Figure 2B. **B**. Graph summarizes maximum tolerated doses (mg/kg) of various classes of chemotherapy agents for targeted weight loss continued on HFD in the absence of tumor challenges. Shaded areas; black = early fatalities, dark gray = weight loss with late fatalities, gray pattern = well tolerated drug with target weight loss, and white = well tolerated drug without target weight loss. Boxed areas denote chemotherapy agents with the greatest non-toxic therapeutic window of targeted weight loss. Results display 3 mice/treatment condition with 3 biological replicates for CY and MTX. **C**. Weekly total body weight measurements showing HFD and LFD treated mice (no tumor) treated with effective doses of MTX or CY. (NB, the greater weight loss of HFD mice receiving CY in Figure 2C compared to Figure 1A is attributable to the higher dose of CY.) Chemotherapy agents were given weekly i.p. for 5 rounds beginning on week 10 (arrows). There were no significant differences in body weights comparing HFD MTX50 and HFD CY200 to LFD without chemotherapy at week 15 (*p*=0.9 and *p*=0.6, respectively, by *t*-test). Significant differences were found at week 15 comparing HFD to LFD (*p*<0.0002), HFD to HFD MTX50 (*p*<0.0006), HFD to HFD CY200 (*p*<0.00007), LFD to LFD MTX50 (*p*<0.002) and LFD to LFD CY200 (*p*<0.02). Each treatment group consisted of 6 mice, each time point showing mean ± SD, data representative of 5 biological replicates. **D**. Normalization of kcal consumed after transitory underfeeding following first chemotherapy dose. Averaged kcal consumed per body weight normalized to calories consumed at start of weekly chemotherapy treatments. There were no significant differences in percent change in kcal consumed/body weight comparing HFD to LFD by *t*-test. Significant difference was observed during TX2 comparing HFD MTX50 and HFD CY20 to HFD (*p*<0.02 and *p*<0.003, respectively) and a significant increase was seen during Post TX comparing HFD MTX50 and HFD CY20 to HFD (*p*<0.03 and *p*<0.02, respectively). Each treatment group consisted of 6 mice, each time point showing mean ± SD, data representative of 2 biological replicates. **E**. Energy was not lost due to malabsorption during chemotherapy. Indirect bomb calorimetry was performed to measure kcals lost in fecal waste before chemotherapy (open bars) and after chemotherapy was completed (filled bars). No significant differences were observed within HFD groups or LFD groups whether or not they received chemotherapy. Each treatment group consisted of 3 mice, each time point showing mean ± SD, data representative of 2 biological replicates.

We sought to determine whether chemotherapy would have similar metabolic impacts in non tumor-challenged, morbidly obese mice, following the schematic in Figure 2A. C57BL/6 mice were weighed before they were segregated into HFD or LFD cohorts. After eight weeks on the respective diets, mice were individually housed and randomly assigned to chemotherapy treatment groups with graded dosing based on whole body weight. Maximum tolerated dose levels of individual chemotherapy agents were established in the non-tumor-bearing setting (irinotecan, paclitaxel, cyclophosphamide (CY), methotrexate (MTX), 5-fluorouracil and gemcitabine) [13].

Mice treated with individual chemotherapy agents presented three different response patterns (Figure 2B): (1) low-end dosing which resulted in insignificant impacts compared to mice not receiving chemotherapy; (2) high-end “toxic” dosing which resulted in persistently decreased food intake, weight loss and inanition whether HFD or LFD; (3) in the cases of MTX and CY, intermediate dosing which, except during the first week of treatment, caused no significant alteration in either continued HFD or LFD food intake, yet paradoxically did cause normalizing weight loss in the obese HFD cohort. The most favorable intermediate doses of weekly MTX (12.5-50 mg/kg) or CY (100-200 mg/kg) proved reliably well tolerated despite their weight reducing impacts (Figure 2C). Dose-response experiments revealed that complete weight normalization could be achieved in non tumor-challenged HFD mice with five weekly doses of MTX50 or CY200 (Figure 2C). Consistent with this phenomenon's dose dependence, slightly lower doses of CY or MTX (e.g., CY100) produced partial weight normalization during that same time period, whether in tumor-challenged or non tumor-challenged HFD mice (Figure 1A and not shown).

We examined the mechanisms by which optimal weekly dosing of MTX or CY accomplished complete weight normalization of HFD obese mice. Preceding chemotherapy treatment, mice fed on HFD nearly doubled in body weight after 10 weeks compared to only a 17% gain in the LFD cohort (Figure 2C). Mice fed on LFD or HFD not receiving chemotherapy continued to gain weight throughout the study (Figure 2C). To assess the mechanisms contributing to weight loss in HFD-fed, chemotherapy-treated mice we measured caloric intake and energy expenditure. Mice fed on either HFD or LFD showed small variability in calories consumed per body weight throughout chemotherapy treatment (Figure 2D). Interestingly, as already observed for tumor-bearing mice (Figure 1B), mice fed on the LFD had a 16% higher caloric intake corrected for body weight compared to mice fed on HFD. HFD-fed mice treated with MTX50 or CY200 experienced transitory underfeeding following the first chemotherapy dose, but caloric intake quickly rebounded to surpass that being consumed by non-treated HFD-fed mice (Figure 2D).

Mice were housed in metabolic cages (CLAMS) for 72h to test possible differences in energy expenditure. The results showed that HFD-fed mice had significantly higher lean body mass energy expenditure compared to LFD-fed mice (caloric value x VO_2_); however, there was no significant difference in lean body mass energy expenditure between the chemotherapy treated and non-treated groups within either the HFD or LFD-fed groups (Supplementary Table S1) [14]. We also observed a modest decrease in activity events in diet-induced obesity (DIO) and even a greater decrease in activity during chemotherapy treatment (Supplementary Figure S1). Therefore, the weight loss observed in obese mice receiving intermediate doses of MTX or CY could not be attributed to either decreased dietary intake or increased metabolic activity.

To examine whether there was a difference in energy excretion, fecal matters from singly housed mice kept in bedding-free cages were collected every 24h during a 3 day collection period. Collected sample weight from all mice, regardless of diet or chemotherapy treatment, averaged 1.7 ± 0.06 g. Bomb calorimetry revealed that there were no significant differences between pre- and post-chemotherapy regarding calories lost fecally in each group, nor were there significant differences between HFD and LFD respective treatment groups (Figure 2E). Therefore, weight loss was not due to chemotherapy-induced malabsorption or persistent loss of appetite. In addition, there was no evidence for ketotic urinary excretion of calories (data not shown).

### Intermediate doses of MTX or CY modulate fat loss in epididymal white adipose tissue (eWAT)

**Figure 3:**
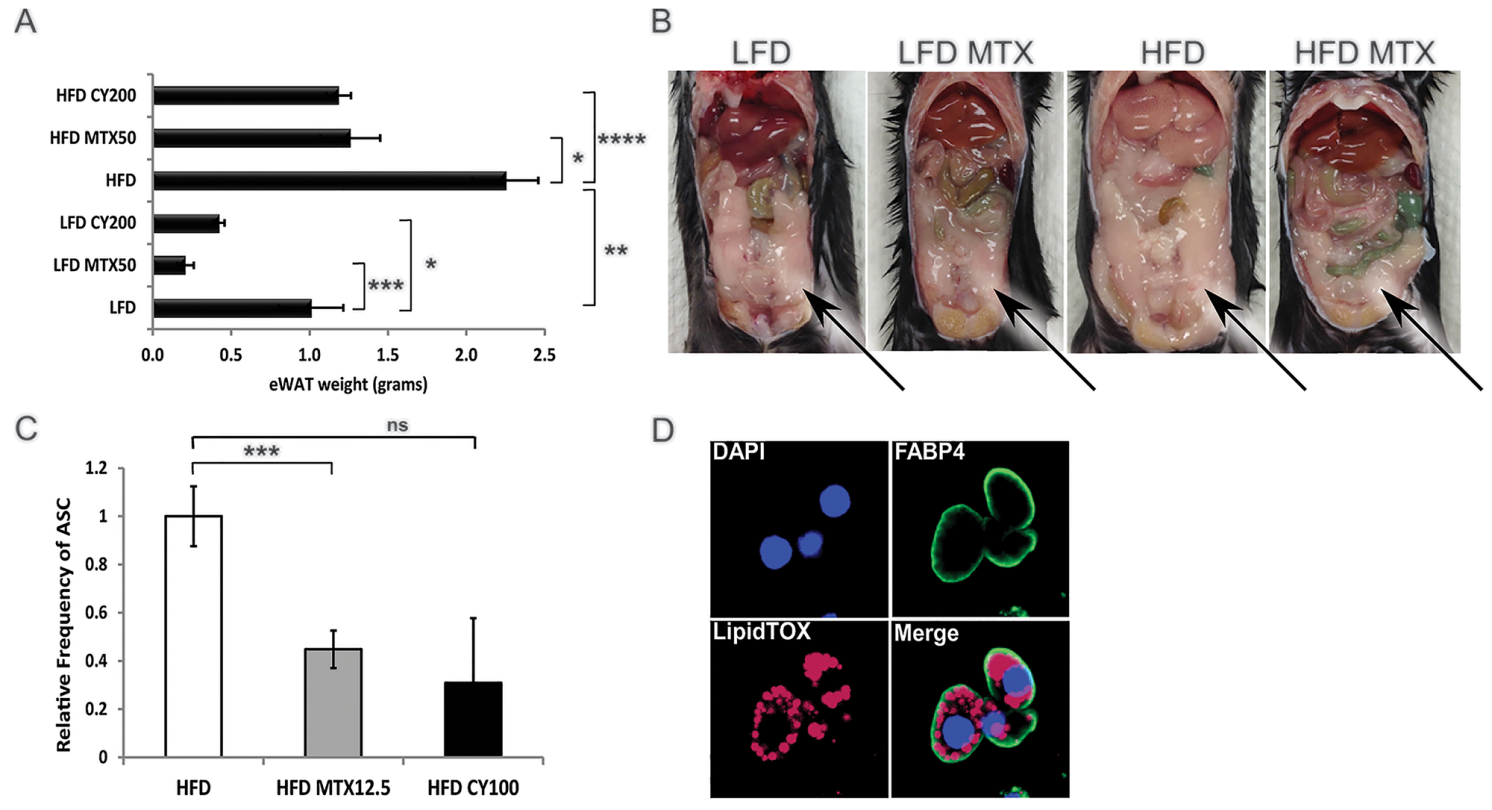
Chemotherapy leads to fat loss in diet induced obese mice **A**. Weight of epididymal white adipose tissue (eWAT) from LFD, HFD, CY200 and MTX50 treated mice 1 week after last chemotherapy treatment. Each treatment group consisted of 6 mice, showing mean ± SD, data representative of 2 biological replicates. Comparisons by Student's *t*-test: ns - not significant, * *p*<0.02, ** *p*<0.004, *** *p*<0.002, **** *p*<0.0007 **B**. Representative illustration of abdominal cavities measured in (A), showing that large visceral fat deposits (arrows) were reduced post chemotherapy in HFD mice. **C**. Relative percent frequency of eWAT-associated adipocyte stem-like cells (ASC) in HFD mice treated with MTX12.5 or CY100. Each treatment group consisted of 3 mice, showing mean ± SD, data representative of 2 biological replicates. Statistics as in (A). **D**. Representative confocal imagery of sorted ASC cultured in adipogenic medium displaying lipid droplets. DAPI (blue)- DNA nuclear stain, FABP4 (green)- lipid transport protein in adipocytes, LipidTOX (red)- neutral lipid stain.

Metabolically active adipose tissue was examined to quantify dietary- and chemotherapy-related alterations in fat storage. The eWAT from HFD mice was determined to be significantly bulkier than in LFD-fed mice (2.2g and 1.0g, respectively, *p*<0.004). When mice were treated with 5-weekly doses of MTX 12.5-50 mg/kg (MTX12.5-MTX50) or CY 100-200 mg/kg (CY100-CY200), eWAT displayed significant reductions: for example, HFD mice receiving MTX50 lost 44% and CY200 lost 48% of eWAT weight compared to non-treated HFD mice, reducing eWAT to levels indistinguishable from LFD mice not receiving chemotherapy (Figure 3A). Predictably, the loss of eWAT in LFD mice receiving chemotherapy was also significant (Figure 3B).

We hypothesized that chemotherapy-induced reductions in eWAT resulted in part from chemotherapy-mediated reductions in adipocyte progenitors [15]. Adipocyte stem-like cells (ASC), conventionally defined as CD31^−^, CD34^+^, CD45^−^ and CD140a^+^ [16], were quantified in resected eWAT from HFD mice, revealing that ASC were significantly reduced (a log-fold in absolute number) by treatment with MTX or CY (Figure 3C, Supplementary Figure S2 and not shown). To verify that these cells were indeed fat-storing precursor cells, they were cultured in adipogenic differentiation medium. Differentiated ASC were then stained with adipocyte fatty acid binding protein 4 (Fabp4), a lipid transport molecule, and neutral lipid LipidTOX and imaged by confocal microscopy to confirm that cells displayed lipid storage potential (Figure 3D). This confirmed that normal ASC are sensitive to chemotherapy agents MTX and CY.

### Intermediate doses of MTX or CY reduce lipid content of fatty liver in non tumor-challenged obese mice

**Figure 4:**
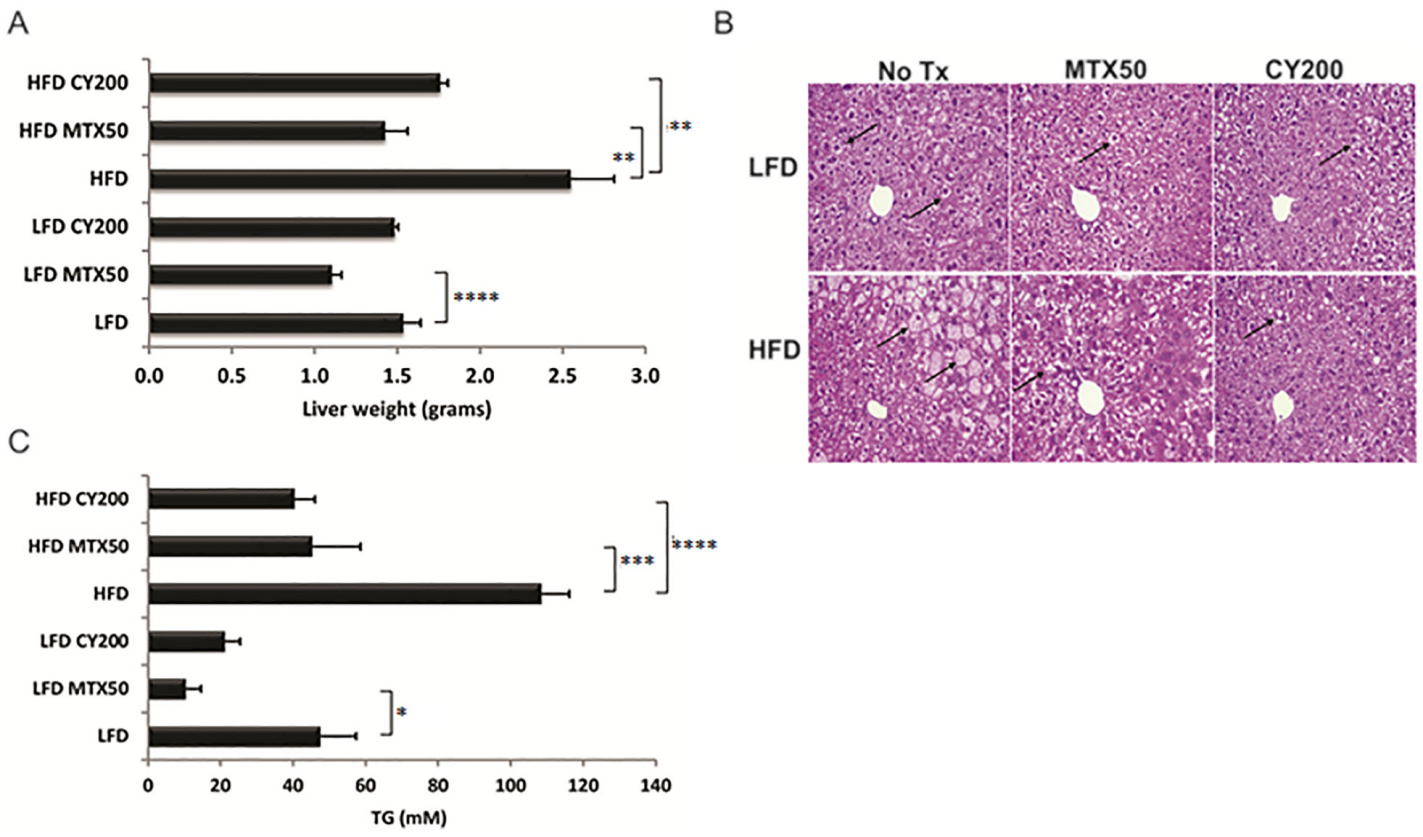
Triglyceride (TG) storage in whole livers is reduced with chemotherapy treatment **A**. Liver weight reduction post chemotherapy treatment. Each treatment group consisted of 6 mice, showing mean ± SD, data representative of 2 biological replicates. As analyzed by *t*-test, * *p*<0.03, ** *p*<0.02, *** *p*<0.008, **** *p*<0.006. **B**. Representative H&E staining of formalin-fixed, paraffin embedded livers displaying reduced lipid content in hepatocytes after chemotherapy treatment (↑ - lipid globules in hepatocytes). **C**. Colorimetric measure of TG levels is significantly lower in flash frozen livers with chemotherapy treatment. Each treatment group consisted of 6 mice, showing mean ± SD, data representative of 2 biological replicates. Significance as in (A).

We examined the consequences of MTX or CY to hepatic metabolism at the same time that these agents reduced adipocyte availability for lipid storage. Without chemotherapy, harvested livers from HFD mice were on average 60% heavier than LFD livers (*p*<0.005). Livers from HFD mice treated with MTX50 or CY200 displayed 44% or 31% (*p*<0.02) average decrease in weight compared to HFD non-treated mice (Figure 4A), effectively normalizing liver weights to those of LFD-fed mice regardless of whether LFD mice also received chemotherapy. Inspection of hematoxylin and eosin-stained liver cross-sections from MTX50 and CY200 treated mice on either diet displayed a parallel reduction in lipid accumulation (Figure 4B).

Intracellular liver triglycerides (TG) were extracted and normalized to liver weight for quantitative comparisons (Figure 4C). Mice fed on HFD without chemotherapy contained significantly elevated levels of TG compared to control LFD mice (108.1 mM and 47.3 mM, respectively). Chemotherapy treated HFD-fed mice stored significantly less TG compared to non-treated HFD-fed mice (MTX50, 44.9 mM and CY200, 40.1 mM, *p*<0.008 and *p*<0.006 respectively). Chemotherapy-induced TG reductions were also seen in the LFD-fed groups (MTX50, 10.3 mM, *p*<0.03 vs CY200, 21.0 mM). These data indicated that optimized doses of MTX or CY facilitated the catabolism and/or clearance of liver TG in HFD-fed mice to the levels observed in LFD-fed mice not receiving chemotherapy.

### Differential gene expression of hepatic lipogenesis relating diet and intermediate doses of MTX or CY in non tumor-challenged mice

**Figure 5:**
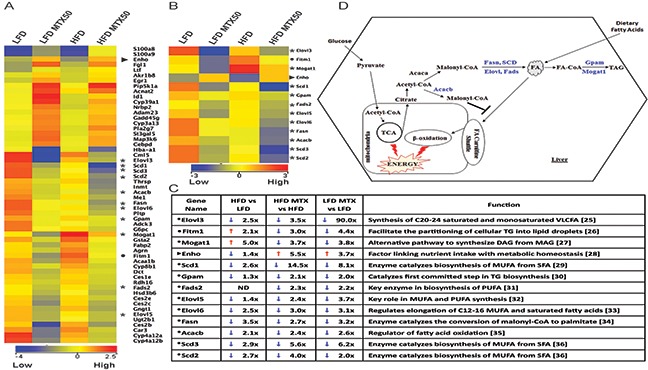
Gene expression analysis of total liver from LFD and HFD diet with and without MTX50 treatment **A**. Heat map of differentially regulated genes (> 2-fold) involved in lipid metabolism. *11 lipogenic genes, ► 1 energy homeostasis gene, • 1 lipid accumulation gene (Blue = lower expression, Red = higher expression). **B**. Lipid metabolism pathway genes > 2-fold change. **C**. Lipid metabolism pathway genes fold-change difference between HFD vs LFD, HFD MTX50 vs HFD and LFD MTX vs LFD [25–36]. **D**. Schematic representation of remodeling of lipid metabolism pathways after MTX50 treatment. Highlighted blue genes are downregulated in HFD and LFD treated with MTX50. MUFA - monounsaturated fatty acids, SFA - saturated fatty acids, TG - triacylglycerol, PUFA - polyunsaturated fatty acids, DAG - diacylglycerol, MAG - monoacylglycerol, VLCFA - very long chain fatty acids, TCA - tricarboxylic acid cycle, ND - no difference.

To further elucidate mechanisms involved in metabolic changes in fatty liver, expression microarray analysis comparing total liver RNA from 4-5 individual male C57BL/6 mice was studied in LFD, LFD MTX50, HFD and HFD MTX50 conditions using Agilent mouse whole genome microarray 4×44K chip set with ~39485 probes. Microarray data were analyzed using GeneSpring 13.0 as described in experimental procedures. Annotated genes (2135) that were found to be differentially expressed when comparing LFD, HFD and methotrexate 50 mg/kg treatment were selected for GeneSpring gene ontology (GO) analysis. There were 113 transcripts differentially regulated in the metabolic process and 55 genes that made the *p*≤ 0.015 cut-off (Figure 5A). Of those transcripts, 13 lipid metabolism-related genes were significantly altered (*p≤* 0.015) in response to HFD and/or methotrexate treatment (Figure 5B).

Key lipogenic enzymes were significantly downregulated in livers after chemotherapy treatment, consistent with suppression of *de novo* fat synthesis (Figure 5C and 5D). Fatty acid synthase (*Fasn*), catalyst of the last step in the FA biosynthetic pathway, was 2.7-fold reduced in MTX50-treated obese mice compared to long-term HFD-fed control mice (Figure 5D). Stearoyl-CoA desaturase (*Scd1*), catalyst of the biosynthesis of monounsaturated FA, showed a 2.6-fold decrease in livers of HFD-fed mice compared to LFD-fed mice. Furthermore, *Scd1* was 14.5-fold reduced in HFD MTX50-treated mice compared to HFD-fed control. Acetyl-CoA carboxylase β (*Acacb*), which catalyzes the carboxylation of acetyl-CoA to the malonyl-CoA pool, was 2.4-fold reduced in chemotherapy-treated mice compared to controls. Elongation of very long chain fatty acids protein (*Elovl*), a family of microsomal enzymes, displayed differential hepatic expression in three of seven isoforms. *Elovl3, Elov5* and *Elov6* were respectively downregulated 3.5-fold, 2.4- fold and 3.0-fold in HFD MTX50-treated mice compared to HFD-fed control mice. Glycerol-3-phospate acyltransferase 1-mitochondrial (*Gpam*), an enzyme on the outer mitochondrial membrane that promotes triglyceride synthesis, was slightly reduced in HFD-fed mice compared to LFD-fed mice; however, when treated with methotrexate, obese mice displayed a 2.1-fold reduction in gene expression compared to HFD-fed controls. Monoacylglycerol acyltransferase 1 (*Mogat1*), an enzyme converting monoacylglycerol to diacylglycerol, was significantly upregulated (5-fold) in obese mice compared to non-obese controls, but when HFD-fed mice were treated with MTX there was a 3.7-fold decreased expression.

These analyses suggested that MTX affects hepatocytes not only by effectively blocking the generation of lipids, but also by impairing the retention of lipids. Fat storage-inducing transmembrane protein 1 (*Fitm1*), a strong promoter of intracellular lipid accumulation [17], was upregulated 2.1-fold in HFD-fed mice compared to LFD, but when treated with MTX50 there was a 3.0-fold and 4.4-fold downregulation in HFD MTX50 and LFD MTX50, respectively. Furthermore, energy homeostasis (*Enho*) gene, known to be down regulated in obesity, hepatosteatosis and insulin resistance [18], was the only gene in the metabolic category showing decreased expression with HFD feeding but an increase in gene expression with methotrexate treatment. Obese mice treated with MTX experienced a 5.5-fold increase in hepatic *Enho* expression, surpassing levels seen in LFD control mice. LFD MTX50 mice also displayed significantly increased *Enho* expression (3.7-fold increase) compared to LFD control.

The particular trends observed for *Enho*, *Gpam* and *Mogat1* in the microarray expression analysis were validated by qRT-PCR (Supplementary Figure S3). Similar metabolic modulations were also observed in HFD-fed and LFD-fed mice treated with CY200 instead of MTX50, although at a reduced intensity compared to MTX50 (data not shown).

With further inspection of the microarray data, it was observed that liver expression of Srebp1 and Mlxipl/ChREBP, key master regulators of lipogenesis, was not significantly affected by prior *in vivo* treatment with MTX50 or CY200, suggesting that these agents’ inhibition of lipogenesis was most likely non-canonical. In addition, hepatic expression of cholesterol metabolism-associated enzymes was not significantly modulated by prior *in vivo* treatment with MTX50 or CY200, effectively decoupling cholesterol metabolism from FA/TG synthesis pathways.

### Methotrexate promotes both ATP production and proton leak in hepatic mitochondria

**Figure 6:**
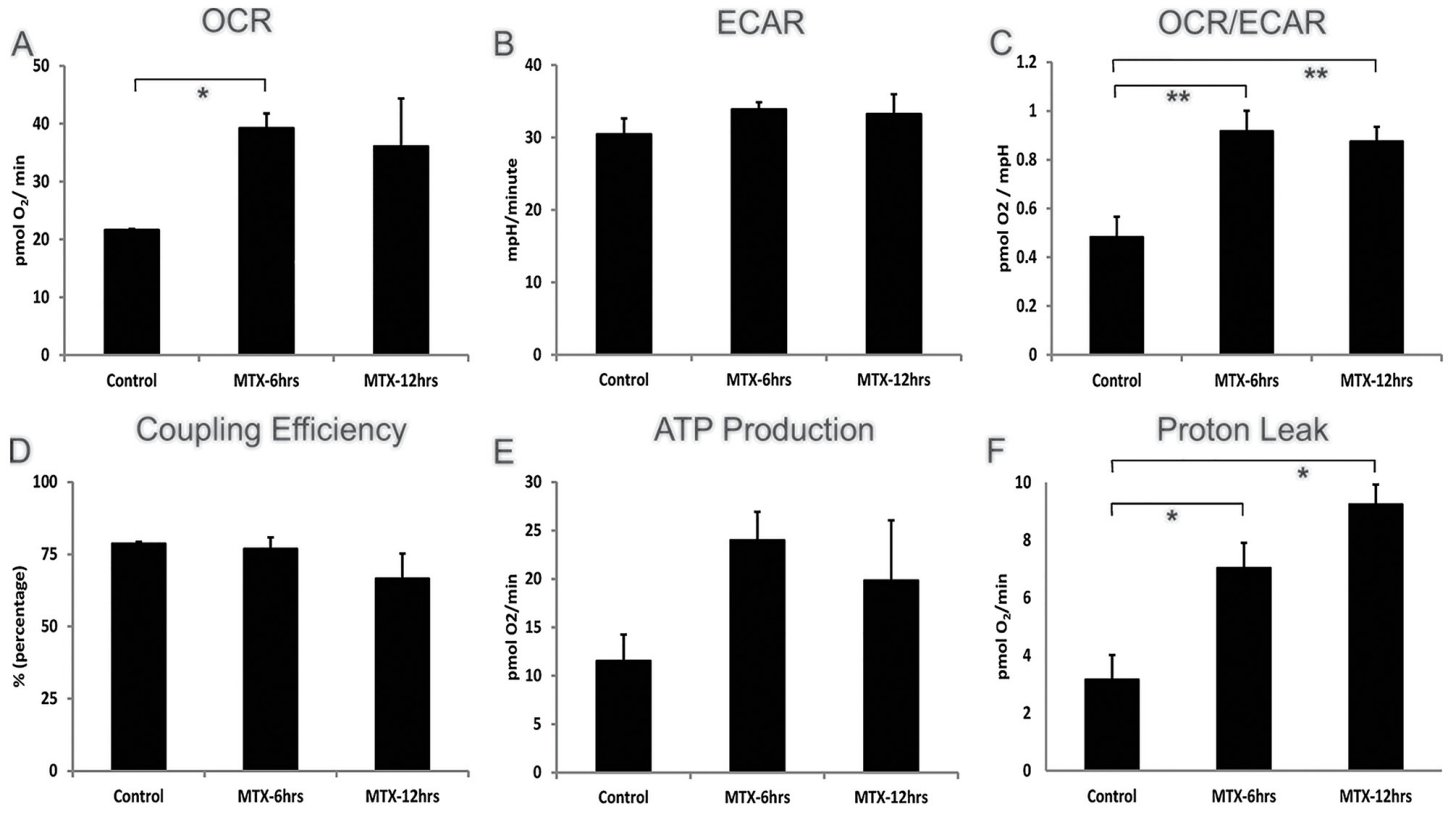
Cellular bioenergetics of human hepatocellular HepG2 cells treated with methotrexate 10 μM Addition of MTX was timed to be 6 or 12hrs prior to the assays performed at the end of a 24hr total culture period. **A**. Steady and increased levels of OXPHOS (basal respiration used to meet a cell's ATP demands) in MTX treated cells, **B**. steady glycolysis (ECAR) rate after MTX treatment, **C**. steady-state OCR/ECAR coefficient ratio continuing during MTX treatment, **D**. consistent coupling efficiency (percentage of basal respiration used to drive ATP synthesis divided by proton leak) in OXPHOS metabolism following MTX treatment, **E**. increased ATP generation (calculated from: OCR basal respiration minus proton leak) in MTX treated cells, **F**. increasing proton leakage (non-ATP linked OCR) across mitochondria inner membrane in MTX treated cells are shown. Each treatment group shows mean of 6 wells ± SD, data representative of 2 biological replicates. *p<0.03, **p<0.05, OCR - oxygen consumption rate, ECAR - extracellular acidification rate, OXPHOS - oxidation phosphorylation, ATP - adenosine triphosphate.

To further investigate our findings of chemotherapy-induced alterations in hepatic metabolism, oxygen consumption rates (OCR), an indicator of mitochondrial respiration, and extracellular acidification rates (ECAR), a measure of glycolytic energy metabolism, were measured in HepG2 cells treated with or without a nontoxic dose of 10 μM MTX. (CY was not tested *in vitro* as its bioactivity requires the generation of active metabolites.) MTX-exposed HepG2 cells showed a significant (MTX-6hrs *p*<0.03) sustained 2-fold increase in OCR (Figure 6A). Furthermore when ECAR was examined, HepG2 cells showed a steady-state rate of glycolysis which was unaffected by MTX treatment (Figure 6B). The OCR/ECAR ratio was determined to measure the relative contribution of mitochondrial respiration and glycolysis to energy management, and it was significantly increased by MTX (Figure 6C). Coupling efficiency, impacted by the mitochondrial respiration rate that drives ATP synthase and the protons that leak across the mitochondria inner membrane (MIM), remained constant during MTX exposure (Figure 6D), due to a sustained significant increase of both ATP production (Figure 6E) and proton leak across the MIM (Figure 6F) during MTX treatment. Such enhanced uncoupling of oxidative phosphorylation (i.e., proton leak) in the face of maintained robust mitochondrial ATP production provides a plausible mechanism to account for chemotherapy-driven weight normalization despite continued ingestion of excessive dietary lipid.

## DISCUSSION

We observed that the growth rate of colon cancer MC38 in male C57BL/6 mice was not significantly impacted by HFD-induced morbid obesity, nor was the tumor's response to chemotherapy, consistent with ASCO guidelines. However, we also observed that chemotherapy agents, especially optimized dosing of MTX or CY, led to a reduction of adiposity and accompanying weight loss in HFD-fed tumor-bearing mice. Moreover, even non-tumor-bearing obese mice with sustained HFD intake normalized their body weight to that of LFD-fed mice during MTX or CY treatments. Finally, cessation of chemotherapy was temporally linked to recommencement of weight gain and recurrent obesity.

Weight loss is a familiar event in cancer patients. Cachexia, a wasting syndrome in which loss of both appetite and body weight occurs involuntarily, can be cancer-induced in the absence of treatment [19], but further aggravated or mimicked by chemotherapeutic agents such as doxorubicin or cisplatin [20–22]. Chemotherapy varies tremendously in its direct metabolic impacts depending upon the agent(s) administered, agent dosing and dosing interval [20–22]. However, apart from chemotherapy's direct effects upon energy metabolism, it is unsurprising that chemotherapy can have antiproliferative impacts upon adipocyte progenitors (ASC) similar to its impact upon bone marrow myeloid progenitors, although this has mostly been modeled with the 3T3-L1 cell line rather than studied in normal ASC [15, 21, 22].

The present report is the first to distinguish a form of chemotherapy-induced weight loss which targets morbidly obese mice, does not require the presence of tumor, and is not associated with such cachexia events as decreased appetite, inanition or foreshortened survival. This form of weight reduction could be rendered entirely normalizing for morbidly obese mice over a well-tolerated five week treatment period, during which time no change in diet or quantity of diet was required. This conglomeration of features was not observed for most of the chemotherapy agents we tested over a range of doses, and in the case of the alkylating agent CY or the antimetabolite MTX only particular dose ranges were both effective and safe (Figure 2B). We confirmed that these effective dose ranges had significant antiproliferative effects upon normal ASC *in vivo*, causing a log-fold reduction in ASC absolute numbers (Supplementary Figure S2B). Remarkably, however, despite the reduced availability of adipocytes for lipid storage, no compensatory increase in hepatocyte lipid storage was observed during MTX or CY treatment, but rather a significant drop in TG storage and lipogenesis.

During the period that HFD mice received five optimized weekly doses of MTX or CY they continued to follow a weight normalization trend which did not reverse until the chemotherapy was terminated, even in non tumor-challenged mice. Therefore, there was no evidence that resistance to chemotherapy developed when normal body cellular constituents were the targets of chemotherapy. Consistent with such lack of resistance, our liver gene arrays for HFD mice demonstrated no significant MTX-attributable downregulation of *Slc19a1* (a main MTX membrane transporter), nor upregulated expression of dihydrofolate reductase (*Dhfr*), a main target of MTX [23].

A key question in the present studies is what becomes of the excessive dietary lard continuously consumed by obese mice receiving MTX or CY, yet accompanied by normalizing weight loss. Common explanations such as malabsorption, increased activity, decreased feeding or diet-induced urinary ketosis were effectively ruled out. Incoming dietary fat storage was thwarted by reduced or unavailable adipocyte storage, due to chemotherapy's depletion of ASC. Despite this additional pressure on the liver to process the continuing glut of incoming dietary TG, CY or MTX treatment was paradoxically also associated with depletion of hepatic lipid stores. In depth analysis of MTX additionally demonstrated this agent's inhibition of hepatic lipogenesis and overall enhancement of mitochondrial respiration, but perhaps most importantly, also revealed a persistence of mitochondrial uncoupling in the face of enhanced overall oxygen consumption, generating a significantly enhanced steady state proton leak. This leak is probably the best candidate mechanism to explain the catabolic dissipation of ingested lipid in a manner which promotes weight loss rather than worsening obesity.

The favorable metabolic effects of MTX or CY chemotherapy upon HFD-fed obese mice is in stunning contrast to the plight of markedly obese patients who achieve impressive weight loss through dietary restraint and intensive exercise, but who subsequently display a poorly understood long-term reduction in their resting metabolism which causes even relatively moderate caloric intake to provoke recurrent obesity [5]. Our findings suggest that MTX, CY and possibly other chemotherapy agents may offer a strategy to address life-threatening morbid obesity in the non-cancer setting. Chronic administration of several chemotherapy agents, including both MTX and CY, is already used clinically to treat non-cancerous diseases such as rheumatoid arthritis, psoriasis and lupus erythematosus. We calculate that the dose of MTX which best counters obesity in mice (12.5-50 mg/kg) is at least a log-fold higher than the dose range given to patients with non-cancerous disease, perhaps explaining why the phenomenon of weight normalization is not apparent as a trend in such patients. Finally, fuller understanding of the mechanisms leading to chemotherapy-modulated normalizing weight loss may identify additional non-chemotherapeutic agents which safely accomplish the same goals.

## MATERIALS AND METHODS

### Animals and animal care

Animal work was performed in accordance of Mayo Clinic Institutional Animal Care and Use Committee. C57BL/6 male mice were purchased from Jackson Laboratories. Starting at 6 weeks of age mice were started on either the control,10% kcal fat energy diet (LFD) or 60% kcal fat energy diet (HFD) (Research Labs, Inc. cat# D12450Bi and D12492i respectively, New Brunswick, NJ) for 10 weeks *ad libitum* prior to treatment or CLAMS (Comprehensive Lab Animal Monitoring System) study. At week 8 on diet mice were housed individually to measure food intake. Mice and food were weighed weekly.

### Cell culture

HepG2 (freshly purchased from ATCC HB-8065, Manassas, VA) and MC38 colon carcinoma (NCI/NIH) cells were cultured in complete growth medium: DMEM (Gibco, Grand Island, NY) supplemented with 10% fetal bovine serum (Gibco), 1000 U/ml penicillin-streptomycin and 2 mM L-glutamine (Gibco) at 37°C/5% CO_2_. Cells were fed every 2-3 days. MC38 cells (5×10^5^) were injected subcutaneously into right flank after 10 weeks on diet. If maximal tumor burden was reached, 2000 mm^3^, mice were euthanized.

### Drugs and treatment

Mice were randomized by body weight and then given weight-based chemotherapy (mg/kg) intraperitoneal (i.p.) x5 weekly injections (except gemcitabine which was given i.p. twice weekly) and tissue harvest was performed 1 week post final treatment. Maximum tolerated doses (MTD) were determined for each of the following chemotherapy compounds: cyclophosphamide (Sigma, St. Louis, MO), 5-fluorouracil (Teva Parenteral Medicines, North Wales, PA ), gemcitabine (Hospira, Inc., Lake Forest, IL), irinotecan (Pfizer, New York, NY), paclitaxel (Hospira, Inc) and methotrexate (APP Pharmaceuticals, Schaumburg, IL) [13].

### Adipocyte stem-like cell (ASC) differentiation

Stromal vascular fraction from epididymal white adipose tissue was isolated using collagenase type VIII (Sigma) digestion. Isolated cells were stained (0.25-0.5 μg/ml per 10^6^ cell in 100 μl volume) with CD31-PE (BD Biosciences cat# 560238, San Jose, CA), CD34-FITC (BD Biosciences cat# 553373), CD45-APC-Cy7 (Biolegend cat# 103116, San Diego, CA) and CD140a-BV421 (BD Pharmingen cat# 562774) and cell sorted with FACS Aria and analyzed with FACS Diva for CD31^−^, CD34^+^, CD45^−^ and CD140a^+^ preadipocytes [16]. Cells were then plated and cultured according to the adipogenic differentiation of mouse mesenchymal stem cells protocol (R&D Systems, Minneapolis, MN). After differentiation, adipocytes were fixed with 4% paraformaldehyde and stained with primary antibody Fabp4 (R&D Systems cat# AF1443, Minneapolis, MN), secondary antibody α-goat Alexa Fluor-488 (ABCAM cat# ab150129, Cambridge, MA), HCS LipidTOX Red neutral lipid stain (Life Technologies, Carlsbad, CA) and Vectashield with DAPI (Vector Laboratories cat# H-1200, Burlingame, CA). Confocal microscopy was performed using Axiovert 200M (Zeiss) with 63x/1.4 oil objective and images analyzed with Zeiss LSM510 software.

### Calorimetry

Minimum of one gram fecal samples were collected for 72h prior to first methotrexate 50 mg/kg or cyclophosphamide 200 mg/kg chemotherapy treatment and for 72h after last treatment. Samples were stored at -80°C until bomb calorimetry was performed by Covance Laboratories (Madison, WI).

### Liver triglyceride measurement

Triglycerides (TG) were extracted from flash frozen liver samples and were measured using the Triglyceride Quantification Kit protocol (Biovision cat# K622-100, Milpitas, CA).

### Histology

Liver sections were fixed in 10% formalin and paraffin embedded (FFPE). 4 micron thick liver samples were stained with hematoxylin and counterstained with eosin (H&E). Microscopy performed using Leica DMRB (20x objective) with Olympus DP71 camera and images analyzed with cellSens Entry 1.12 software.

### RNA extraction and microarray processing

RNA was isolated using RNeasy mini kit (Qiagen cat#74104, Hilden, Germany) according to manufacturer's instructions. RNA quantity and integrity were verified with the Nanodrop (Thermo Scientific) and Bioanalyzer using the Nano chip (Agilent). Using the Quick Amp Labeling kit, one color (Agilent), 200 ng RNA per sample was labeled. Dye incorporation and amplified RNA amounts were verified with Nanodrop. Then, 1.65 μg of each sample was hybridized to a 4×44K mouse whole genome slide (Agilent), washed, and feature extracted according to manufacturer's (Agilent) instructions.

### Microarray data analysis

Gene expression imaging, quality control, fold change, GO (gene ontology), and clustering analysis was performed with GeneSpring GX 13.0 (Agilent Technologies, Inc.). Differentially regulated gene lists, 2-fold change or greater, were selected between LFD and HFD and between methotrexate treated and non-treated mice. Metabolic process list was generated using GO analysis with p-value≤0.05. Hierarchical clustering analysis was performed using gene lists that were differentially regulated with a *p*-value ≤0.015 rather than 0.02 for greater stringency [24]. Every treatment cohort consisted of 4-5 biological replicates. The data have been deposited in NCBI's Gene Expression Omnibus and are accessible through GEO Series accession number GSE87729.

(https://www.ncbi.nlm.nih.gov/geo/query/acc.cgi?acc=GSE87729)

### Comprehensive lab animal monitoring system (CLAMS)

Mice fed on LFD or HFD for 10 weeks prior to placement into a 12 chamber Oxymax/CLAMS (Columbus Instruments, Columbus, OH) with free access to food and water. Mice were allowed to acclimate in individual metabolic cages at 22°C for 16h before measurements were taken. 72h metabolic profiles were generated in successive 26min cycles. Oxygen consumption, CO_2_ production, respiratory exchange ratio (RER), food intake and spontaneous locomotor activity were measured.

### Measurement of mitochondrial function and oxidative stress of HepG2 cells

Human hepatocellular HepG2 cells were untreated or treated with MTX 10 μM for either 6h or 12h prior to extracellular flux analysis. HepG2 cells were washed with PBS and cells were seeded at 40000 cells/well in XF96-well cell culture microplate (Seahorse Bioscience, North Billerica, MA). Seahorse Bioscience XF96^e^ Extracellular Flux Analyzer was used to monitor the oxygen consumption rate (OCR, XF Cell Mito Stress Test Kit) and extracellular acidification rate (ECAR, XF Glycolysis Stress Test Kit) in intact HepG2 cells.

### Statistics

Within individual experiments, each treatment condition (e.g. HFD vs LFD with or without chemotherapy) was tested in individual groups of mice to determine means ±SD for various parameters at particular time points, such as weight or kcal consumed. Such groups were compared via unpaired Student's *t*-test, with *p*≤ 0.05 considered significant. In Seahorse experiments, 6 wells were concurrently performed and similarly analyzed for each condition by Student's *t*-test. Therapeutic impact of diet and chemotherapy upon tumor growth was analyzed in a binary fashion (permanent tumor eradication, yes or no) by Fisher's exact-test. Statistical analyses of microarrays are described above.

## SUPPLEMENTARY FIGURES AND TABLE



## Abbreviations

ASC: adipocyte stem-like cells
ASCO: American Society of Clinical Oncology
CLAMS: comprehensive lab monitoring system
CY: cyclophosphamide
DIO: diet-induced obesity
*Enho*: energy homeostasis (gene)
eWAT: epididymal white adipose tissue
GO: gene ontology
HFD: high fat diet
LFD: low fat diet
MTX: methotrexate
TG: triglycerides
