# Supplementary material for: Chemotherapy can induce weight normalization of morbidly obese mice despite undiminished ingestion of high fat diet
Source: Oncotarget. 2017 Jan 10;8(3):5426–38. doi: 10.18632/oncotarget.14576 (PMC5354920; doi:10.18632/oncotarget.14576)
Supplement: Supplementary file 1 [file oncotarget-08-5426-s001.pdf]

Chemotherapy can induce weight normalization of morbidly obese mice despite undiminished ingestion of high fat diet

SUPPLEMENTARY FIGURES AND TABLE

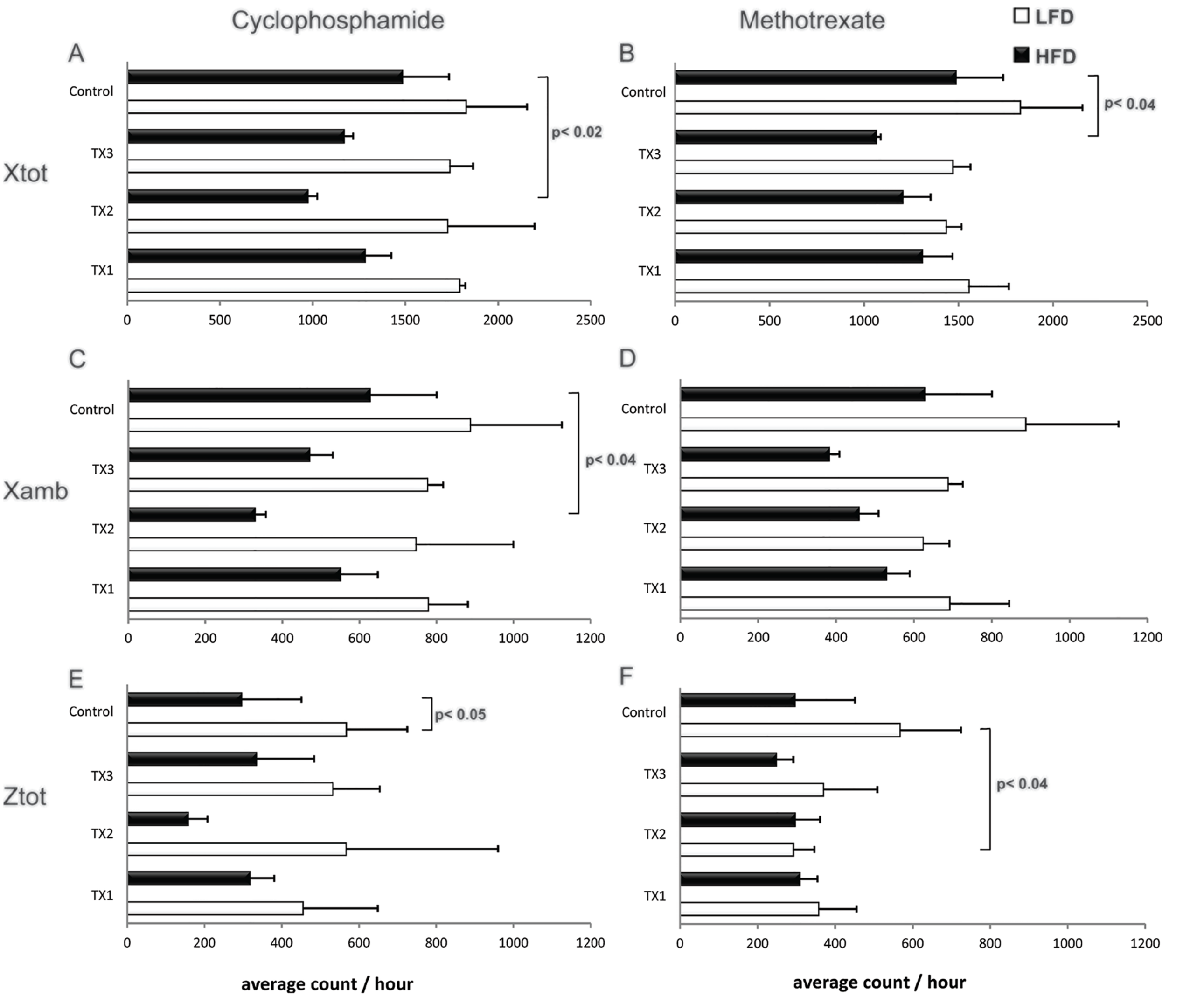

Supplementary Figure 1: Average motor activity of mice on various chemotherapeutic agents. HFD CY100, HFD MTX12.5, LFD CY100, LFD MTX12.5 and control cohorts HFD and LFD were counted after each of the 3 cycles of chemotherapy (cyclophosphamide 100 mg/kg and methotrexate 12.5 mg/kg). Infrared beam interruptions in both horizontal (X) and vertical (Z) directions were used to quantify the motor activity of mice in CLAMS units. Any horizontal beam breakage was recorded as total activity count (Xtot), two or more consecutive horizontal beam breakages were recorded as ambulatory activity count (Xamb). Any vertical beam breakage was recorded as total activity count (Ztot). Decrease in activity levels in HFD-fed mice compared to LFD and HFD CY100 **A, C, E.** and HFD MTX12.5 **B, D, F.** shows lower activity than control cohort. Significant differences are identified in the graphs.

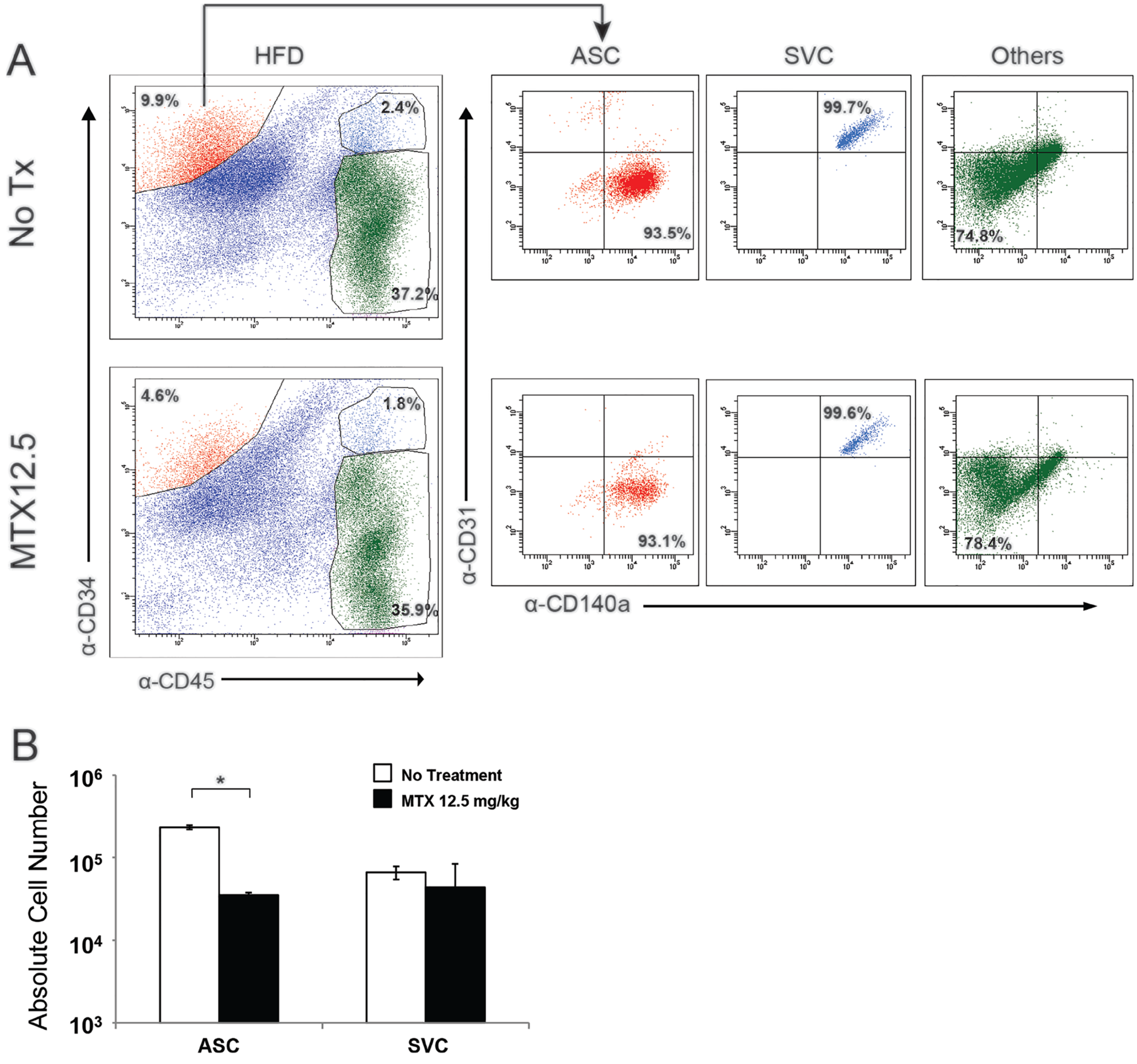

Supplementary Figure 2: Reduction of adipocyte stem-like cells in eWAT after 5 cycles of methotrexate (12.5 mg/kg) from HFD-fed mice. A. Representative flow cytometry dot plots show a decrease in ASC (red gate). ASC - adipocyte stem-like cells (red: CD34+CD45-CD31-CD140a+), SVC - stromal vascular cells, non-adipogenic cells (light blue: CD34+CD45+CD31+CD140a+), Others (green: CD34+CD45+CD31+CD140a-). Data show that, in addition to the overall decrease in cell numbers in eWAT following chemotherapy (Figure 3A), the relative frequency of ASC within eWAT also decreased (left panes Supp. 2A). Right panel Supp. 2A: with or without chemotherapy treatment, the CD34+CD45- fraction (red) consists of highly pure ASC (93.1% vs 93.5% CD31-CD140a+). **B.** Absolute number of eWAT-associated ASC decreases. bit not SVC, during treatment with MTX (shown in log scale). * p<0.002, No Tx - No treatment, MTX12.5 - methotrexate 12.5 mg/kg.

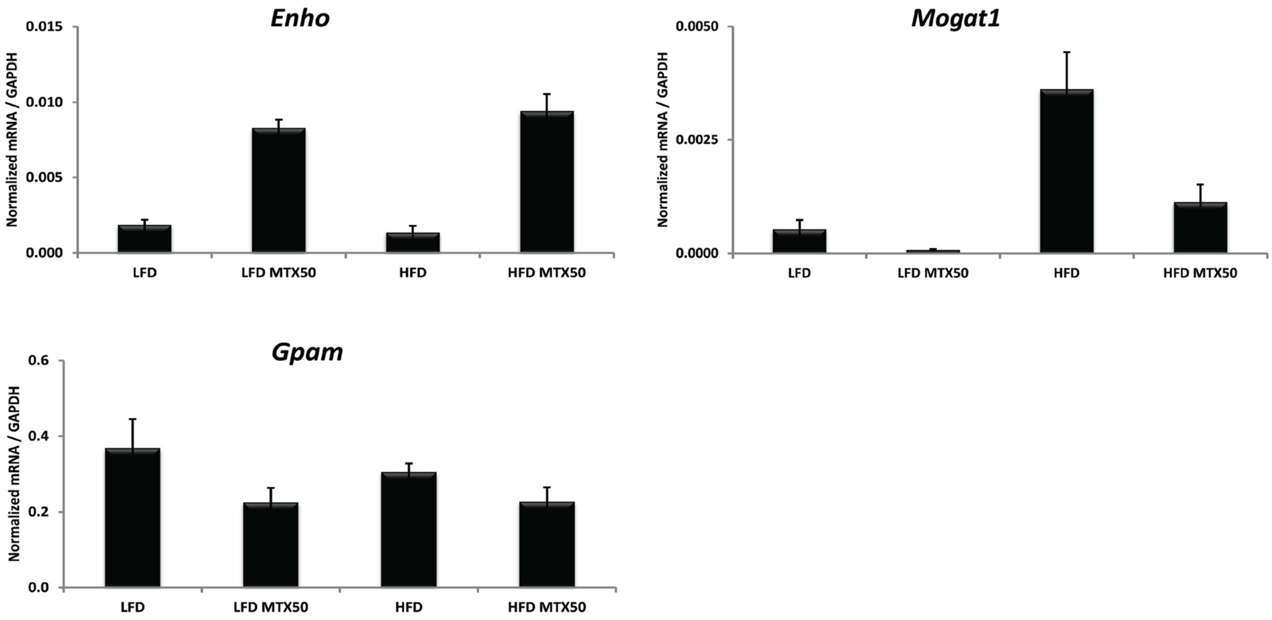

Supplementary Figure 3: Validation of gene expression by qR*T*-PCR. Total RNA isolated for microarray analysis was reverse transcribed with random hexamer primers using Superscript III (Invitrogen). The resulting cDNA was amplified by PCR using LightCycler FastStart DNA Master Plus SYBR Green (Roche) and the following gene-specific primers (Operon); Gapdh-F 5'-accacagtccatgccatcac-3', Gapdh-R 5'-tccaccaccctgttgctg*t*-3', Enho-F 5'- ctcatcgccatcgtctgcaa*t*-3', Enho-R 5'-gggactggattccgagagaga-3', Gpam-F 5'-acagttggcacaatagacgtt*t*-3', Gpam-R 5'-ccttccatttcagtgttgcaga-3', and Mogat1-F 5'-agcgcaaagggtttgttaag-3', Mogat1-R 5'-cagcttccgatagggcatta-3'. The cDNA amount in each sample was normalized to the crossing point of the housekeeping gene Gapdh. Relative mRNA fold change for each gene was calculated using the respective crossing points applied in the following formula: F = 2−(G − H), where F = fold difference, G = gene of interest, and H = housekeeping gene (modified from Hoelzinger et al. J Neurooncol 2008; 86: 297-309).

Supplementary Table 1: Lean body mass energy expenditure (kcal/day) for mice on various chemotherapy and diet regimens

Diet Comparison		Intercept Estimate	StdError	p Value
HFD	LFD	26.75	9.99	0.044
HFD	HFD MTX12.5	24.94	10.78	0.082
HFD	HFD CY100	17.56	12.98	0.247
LFD	LFD MTX12.5	13.85	11.48	0.294
LFD	LFD CY100	16.07	12.25	0.26
HFD MTX12.5	LFD MTX12.5	10.16	12.86	0.487
HFD CY100	LFD CY100	-7.66	11.08	0.539

Mice receiving HFD, LFD, HFD MTX12.5, HFD CY100, LFD MTX12.5 and LFD CY100 were compared using EE ANCOVA analysis [14]. Energy expenditure is significantly up regulated in HFD-fed mice compared to LFD. No significant difference in energy expenditure is seen between the chemotherapy treated and non-treated groups within either the HFD or LFD-fed groups.
